# A Systems Factorial Technology dataset using visual and tactile cues to guide balance

**DOI:** 10.1016/j.dib.2019.104573

**Published:** 2019-09-26

**Authors:** Devin Burns

**Affiliations:** Missouri University of Science and Technology, USA

**Keywords:** Systems factorial technology, Workload capacity, Balance

## Abstract

This data contains response times for 19 participants from a Systems Factorial Technology paradigm using visual and vibratory cues, as described in “The Balance Between Vision and Touch [1]”. These cues could indicate one of four directions, and participants responded by shifting their weight in that direction. This was detected using a Wii Balance Board. Each participant has 720 trials: 1/3 with only a haptic cue, 1/3 with only vision, and 1/3 with both. Cues were equally divided into high and low salience versions.

**Table:** Specifications Table

Subject area	*Psychology*
More specific subject area	*Cognitive/Mathematical Psychology*
Type of data	*CSV files of trial by trial response time and accuracy*
How data was acquired	*An Arduino Mega received signals from a Wii balance board and recorded Center of Pressure data to determine responses.*
Data format	*Raw*
Experimental factors	*Direction of cue, salience (or absence) of each cue modality*
Experimental features	*There were 6 blocks of 60 trials for each of 2* 30-min *sessions for 19 participants*
Data source location	*Rolla, MO, USA*
Data accessibility	*In OSF repository:*https://osf.io/chpqr/
Related research article	*Burns, D. M. (in press). The Balance Between Vision and Touch. Journal of Mathematical Psychology* [1].

**Table:** 

**Value of the data** • This data is suitable for Systems Factorial Theory (SFT) analysis for probing mental processing structures • SFT researchers can use this data for model testing and verifying new analyses • This experiment uses a novel “lean-to-respond” methodology that would be valuable to compare with traditional response-pad data • This data would be useful for examinations of the effect of base time, as the response modality takes longer to execute

## 1 Data

This data contains trial-by-trial response times and accuracy for nineteen participants in a redundant targets experiment where visual and or tactile stimuli cued responses which were provided by leaning in one of four directions on a balance board. Raw data files are provided with response time, stimulus salience, and trial/block number for each participant and session. Processing code and an aggregated, cleaned data set are also provided, along with analysis code for SFT analyses and a hierarchical Bayesian analysis following [2].

## 2 Experimental design, materials, and methods

This experiment employed the double factorial paradigm, where two different cues (one visual and one haptic) could either be high salience, low salience, or absent, yielding eight stimulus combinations (there were no absent-absent trials). Nineteen participants were recruited with the only participation criteria being that they were 18 or older and have no balance problems. All participants provided informed consent as approved by the Internal Review Board. Participants were asked to return for a second 30 minute session within seven days, but only 11 did so. All participants were compensated with $5 for each session they completed.

For the vibrotactile feedback, we designed and built an adjustable belt with four vibration motors positioned around it on the left and right sides of the front and back of the body. This belt was connected via bluetooth and controlled by an Arduino micro-controller, which was synchronized with a computer connected to a CRT monitor. This monitor displayed experimental instructions and feedback, as well as displaying visual cues in the form of small red circles. These circles appeared in one of the four corners of a white square in the middle of the screen, with the top left corner signifying that participants should lean forward and to the left. Participants were positioned 66 cm from a CRT monitor such that the white square subtended approximately 8° of visual angle, with the circles sized at 1°. In order to balance cue strength, high salience visual cues were displayed at only 8% opacity, while low salience were displayed at 3%. High salience vibrations were driven at 5 V and low were driven at 2 V.

Participants were then shown a demo screen that displayed how their CoP moved when they shifted their weight. When they were ready to proceed, they were given 16 practice trials, two of each possible stimulus combination. If they had no further questions, the experimenter left the room and they proceeded with the first of six blocks of trials.

Each block had 60 trials: 20 redundant trials where both types of cues were presented, and 20 single target trials with only a visual cue, and 20 trials with just a haptic cue. The trials were distributed in this way such that the presence of one cue carried no statistical information regarding the presence or absence of the other. The trials were then randomly shuffled within each block. The direction indicated by the cue was determined randomly for each trial, and for redundant trials both cues always indicated the same direction.

On every trial, a fixation cross was displayed for a randomly generated time between 500 and 1000 ms. After this time, a cue was presented either on screen, through the belt, or both. Cues remained present for 3 seconds or until a response was issued. If no response was detected within the 3 seconds, a “time out” was declared and the next trial began. Responses were determined by averaging the weight at each corner within a moving window containing the most recent 20 samples taken at 100 hz (so 200 ms). A response was declared when the weight at one corner increased by 10% above its baseline value, and the response time was recorded as the elapsed time since the cue was turned on. In the chance that two corners both increased above the threshold at the same time, the one that increased more was deemed to indicate the response.

Data for all participants is included even those who only completed one session, as well as code for processing and cleaning the data. More procedural and analysis details can be found in Ref. [1].

## References

[1] Burns D.M. (2019). The balance between vision and touch. J. Math. Psychol..

[2] Houpt J.W., Heathcote A., Eidels A. (2017). Bayesian analyses of cognitive architecture. Psychol. Methods.

